# Correlates of Alcohol Consumption Among a Socially-Disadvantaged Population in Poland

**DOI:** 10.3390/ijerph17239074

**Published:** 2020-12-04

**Authors:** Kinga Polanska, Dorota Kaleta

**Affiliations:** Department of Hygiene and Epidemiology, Medical University of Lodz, 90–752 Lodz, Poland; dkaleta@op.pl

**Keywords:** inequalities, alcohol consumption, lifestyle factors, healthy lifestyle index, correlates, socially-disadvantaged population

## Abstract

Alcohol consumption at a level exceeding existing recommendations is one of the leading risk factors for death and disability worldwide. The aim of the study was to identify correlates of alcohol drinking among a socially-disadvantaged population in Poland. The cross-sectional study covered 1644 adult social assistance beneficiaries from the Piotrkowski district (rural area in central Poland). A detailed questionnaire filled in during a face-to-face interview allowed for the collection of socio-demographic, lifestyle-related (including alcohol consumption) and health status data. About 42% of the participants, including 67% of the men and 30% of the women, exceeded the recommended level of alcohol consumption. In the adjusted model, the men tended not to follow recommendations for alcohol consumption more frequently than the women (OR = 4.5, *p* < 0.001). The higher odds of not following alcohol-related recommendations were also observed for the subjects declaring having a permanent or temporary job compared to the unemployed participants (OR = 1.2, *p* = 0.04). A lower healthy lifestyle index (indicating an unhealthy lifestyle related to a diet, body mass index (BMI), physical activity, and tobacco smoking) was associated with not following recommendations for alcohol consumption (OR = 1.1, *p* = 0.04). Our study indicates that being men, having a permanent or a temporary job, and coexistence of other unfavorable lifestyle-related factors are important correlates of not following recommendations for alcohol consumption among the beneficiaries of government welfare assistance.

## 1. Introduction

Alcohol use is recognized as an important determinant of health. Reducing alcohol drinking might aid in achieving some of the 17 Sustainable Development Goals (SDG) adopted by all United Nations Member States in 2015, including those focusing on maternal and child health, non-communicable diseases (NCD), infectious diseases as well as mental health, injuries, and poisonings [1,2,3,4]. Alcohol consumption is also shown as one of the indicators for the SDG health target 3.5—“Strengthen the prevention and treatment of substance abuse, including narcotic drug abuse and harmful use of alcohol” [2].

In 2016, about 2.3 billion people (43%) of the global population aged 15 and older were current drinkers [3]. Moreover, the highest levels of alcohol consumption are still observed in the countries of the World Health Organization (WHO) European Region. According to the WHO Global Health Observatory in 2015–2017 period, in Poland, the average total (recorded and unrecorded) alcohol consumption was 11.6 L of pure alcohol per person aged 15 or over, and it was 0.2 L more than during the 2009–2011 period [3].

Alcohol drinking contributed to 3 million deaths (5.3% of all deaths) worldwide and 132.6 million (5.1%) disability-adjusted life years (DALYs), including 2.3 million deaths and 106.5 million DALYs among men [3]. Of all alcohol-related deaths, about one-third was due to injuries, which is the same proportion due to cardiovascular diseases or cancers, one-fifth due to digestive diseases, and 13% due to infectious diseases. About one-half of alcohol-attributable DALYs were due to NCD and mental health conditions. It needs to be pointed out that the proportions of all deaths and DALYs attributable to alcohol consumption were also the highest in the WHO European Region (10.1% of all deaths and 10.8% of all DALYs) [3].

Numerous studies have confirmed that lifestyle-related behaviour, health, and well-being are influenced by individuals’ socio-economic factors. Although, in recent years, improvement in living standards have been observed in the majority of the developed countries, while disparities between or within a country are still widening [1,5,6]. Education, employment, and income are the most frequently pointed out determinants related to socio-economic status (SES) that can influence both patterns of alcohol consumption and their health-related harm. This has been confirmed by the literature reviews, which indicate complex, multifaceted relationships between social determinants, inequities, and alcohol consumption [5,6], on one hand, and the role of alcohol use and drinking patterns in socio-economic inequalities in mortality, on the other hand [1,7,8,9]. However, individuals with a higher SES may consume similar or greater amounts of alcohol compared to those with a lower SES. The latter group seems to bear a disproportionate burden of negative alcohol-related consequences [1,7,8,9,10,11]. This phenomenon can result from the fact that other lifestyle-related factors such as tobacco smoking or obesity cluster in individuals with a low SES and interact with alcohol use, resulting in elevated risks related to alcohol use in this group [12]. Access to healthcare services as well as variations in drinking patterns are additional potential factors contributing to the exacerbated health consequences of alcohol use among individuals with a low SES [13,14].

Taking into account the above considerations, understanding the correlations between the variety of socio-demographic and lifestyle-related factors and alcohol consumption among people representing an unfavorable economic situation is critically important for identification and development of effective interventions. To the best of our knowledge, such comprehensive analyses have not been performed among such vulnerable populations in Poland. Thus, the aim of this study was to evaluate the correlates of not following recommendations for alcohol drinking among social assistance beneficiaries from a socio-economically disadvantaged rural area in Poland.

## 2. Material and Methods

### 2.1. Study Design and Population

The series of analyses, including correlates of tobacco smoking, diet, and recreational physical activity among socially-disadvantaged adults (18–59 years) from the Piotrkowski district (central Poland) have been already published [15,16,17]. According to the data from 2013, there were 91,618 residents living on the premises of the district with more than 90% of them representing rural areas. Approximately 9% of its residents required the support of social assistance institutions due to the lack of resources to live on. An analysis completed by the United Nations Development Program (UNDP) identified the district as the 11th among all 314 rural districts with the lowest indicators of social development in Poland. The Local Human Development Index (LHDI), covering three indicators: Health Index, Education Index, and Welfare Index, was one of the lowest in the country [15,16,17].

Our cross-sectional study covered residents of the Piotrkowski District registered with the local government welfare assistance institutions and entitled to receive social aid. The poverty threshold (a monthly income equal to or less than 634 PLN (148 Euro) for a single person or 514 PLN (120 Euro) for a family member) has been used by the social assistance institutions to ensure social aid according to the regulations specified in The Social Welfare Act [18,19]. This poverty threshold is approximately 50% of the minimal net salary. Following the inclusion criteria (age and income restrictions), 3636 people were eligible for the study, 1817 (50.0%) individuals agreed to participate in the study, and 1644 participants (90% of those who filled in the questionnaire) provided data about alcohol consumption crucial for the current analyses.

The study was approved by the Bioethics Committee of the Medical University in Lodz (RNN/243/15/KE).

### 2.2. Alcohol Consumption

The detailed information on alcohol consumption was obtained using a questionnaire that was filled in during an interview conducted by trained staff at the place of residence of each participant. The respondents declared frequency (never, rather than once per month, average times per month, average times per week, everyday) and intensity (average volumes of alcohol per one occasion) for each type of drunk alcohol (spirit, beer, and wine). Based on this information, a unit of alcohol per occasion and per week was calculated following the formula: unit of alcohol = (volume (ml) × abv)/1000. The participants were also asked about the frequency of binge drinking described as five or more drinks of alcohol on one occasion (never, rare (1–2 times per year), once per month, once per week, few times per week). It is proven by the existing research that frequent and high-quantity alcohol consumption (exceeding daily and weekly limit and binge drinking) is related to a poorer health-related quality of life [3,20,21]. The following three recommendations were considered: 1) daily limit—not more than 40 g of pure alcohol for men (4 units) and 20 g of pure alcohol for women (2 units), 2) weekly limit—not more than 210 g of pure alcohol for men (21 units) and 140 g of pure alcohol for women (14 units), and 3) no binge drinking (5 or more drinks per occasion at least once per month). Thus, a participant was defined as following alcohol-related recommendations if all of the three previously indicated recommendations were followed.

### 2.3. Corelates of Alcohol Consumption

As described previously, information about socio-demographic and lifestyle characteristics as well as health status of the participants was collected by using the detailed questionnaire [15,16,17,22]. The following socio-demographic variables were selected for the current study: the participants’ sex, age, educational level, employment status, subjective assessment of monthly income, living conditions and life satisfaction, cohabitation status, and presence of children below 15 years of age in the household. The participants were also asked several questions dedicated to the following lifestyle-related characteristics: smoking, height and weight, diet, and recreational physical activity. The current smoking status was evaluated based on a question “Are you currently smoking cigarettes?” (the participants who answered “no” were classified as following smoking-related recommendations) [22]. Based on height (m) and weight (kg), body mass index (BMI) (kg/m^2^) was calculated. Healthy weight was defined as BMI between 18.5 and 24.9 (kg/m^2^) (thus, the participants were considered as following BMI-related recommendations) [23]. The study participants reported the frequency of fruit and vegetable consumption. The individuals who consumed eight or more combined servings of fruits and vegetables per day were considered as following diet-related recommendations [24]. Finally, the study subjects were asked to indicate frequency (per week), intensity (moderate-intensity physical activity and vigorous-intensity physical activity), and length (minutes per day) of recreational physical activity. It is recommended that adults participate in 150 minutes per week of moderate-intensity physical activity, 75 minutes per week of vigorous-intensity physical activity, or 115 minutes of combined-intensity physical activity per week [25]. The participants who achieved at least one of the previously mentioned criteria for the recommended level of recreational physical activity were coded as following recommendations related to recreational physical activity.

As described by Znyk et al., 2020, for each of the four lifestyle-related characteristics (smoking, BMI, diet, and recreational physical activity), the participants could receive 1 if they followed the relevant recommendations and 0 if the recommendations were not followed [22]. Based on this data, the combined healthy lifestyle index (HLI) was calculated as the sum of points given for each of the four lifestyle-related factors. This index can range from 0 to 4, were 4 means that a participant follows all the analyzed lifestyle-related recommendations. The participants’ health status was evaluated using two questions: “How can you describe your health state?” (with the following possible answers: fair, rather fair, neither fair nor poor, rather poor, poor) and “Have you noted the following health problems within the past month?” (with yes/no option for the following health problems: chest pain, joint pain, back pain, headache, insomnia, severe depression, hypertension, gastrointestinal problems, other—please specify).

### 2.4. Statistical Analysis

For the variables included in the study, the numbers and percentages (as a proportion of the study sample or selected category) were presented.

The analysis of not following recommendations for alcohol consumption by socio-demographic and lifestyle-related characteristics of the population was performed. The unadjusted and adjusted odds ratios (OR), 95% confidence intervals (95% CI), and *p*-values were calculated. The multivariable logistic regression was performed with the inclusion of all of the studied variables with *p*-values below 0.1 in the univariable analyses. The analyses stratified by gender were also presented in Supplementary Materials. The standard significance level *p* < 0.05 was selected for the interpretation of the results. STATISTICA version 10.0 (Dell Software, Arizona, CA, USA) was used to perform the statistical analysis.

## 3. Results

### 3.1. Characteristics of the Study Sample

Characteristics of the study sample are presented in Table 1. The study covered more women than men (67%), mostly the respondents in the age category between 30–49 years (76%), with a lower educational level (60%) and unemployed (58%). Most of the participants described their health state as fair or rather fair (65%). About 37% of the beneficiaries of government welfare assistance declared a smoking status. Additionally, the proportion of the participants who did not follow recommendations related to a healthy diet (90%), recreational physical activity (74%), or BMI (58%) was high. As a consequence, the combined healthy lifestyle index equal 0 (which means that all four lifestyle-related recommendations were not followed) was observed in 18% of the study sample and equal 1 (which means that only 1 recommendation was followed) in 40% of the participants. Only 18 individuals (1.1%) received the maximum number of points for a combined healthy lifestyle index.

### 3.2. Correlates of not Following Recommendations for Alcohol Consumption

About 42% of the participants, including 67% of the men and 30% of the women, exceeded the recommended level for alcohol consumption (Table 1). In the adjusted model, the men tended not to follow recommendations for alcohol consumption more frequently than the women (OR = 4.5, *p* < 0.001) (Table 2). The higher odds of not following alcohol-related recommendations was also observed for the subjects declaring having a permanent or temporary job compared to the unemployed participants (OR = 1.2, *p* = 0.04). A lower combined healthy lifestyle index was associated with not following recommendations for alcohol consumption (OR = 1.1, *p* = 0.04). In other words, the individuals who declared no healthy lifestyle-related behaviour (regarding a diet, BMI, physical activity, and smoking) at the same time did not comply with the recommendations on alcohol consumption. Table S1 presents results of the analyses stratified by gender. Only the men who declared absence of the children below 15 years of age in the household consumed alcohol at the level exciding the existing recommendations more frequently than those having children (OR = 1.5, *p* = 0.06).

## 4. Discussion

Our study indicates that, even in a rather homogenous population of government welfare assistance beneficiaries from one region of Poland, some differences in alcohol consumption exist. Higher odds of not following recommendations for alcohol consumption was observed among the men, subjects declaring having a permanent or a temporary job, and those with other unfavorable lifestyle-related factors. These results are crucial for the development of effective interventions that take into consideration specific needs of disadvantaged individuals.

Our analyses indicate that about 42% of the beneficiaries of government welfare assistance, including 67% of the men and 30% of the women, exceeded the recommended level of alcohol consumption. An advantage of the selected approach to evaluate if the study subjects followed alcohol-related recommendations (as a complex indicator) as compared to evaluating drinking status, average volume of alcohol consumption, or binge drinking separately, is that, this way, we have a full picture of an individual’s drinking pattern. However, such an approach limits direct comparison of the level of alcohol consumption in our study with that of the general population in Poland or with data from other countries. The existing analyses indicate that alcohol consumption in Poland has remained at a level close to the average consumption in Europe. However, higher prevalence of alcohol drinking can be observed among disadvantaged groups [3]. Binge drinking or heavy episodic drinking (HED), usually defined as drinking at least 60 g or more of pure alcohol on at least one occasion at least once per month (which equals to 5 or more drinks per occasion at least once per month as selected in the current study), is of specific concern considering its public health and clinical consequences [3,21]. In our study, binge drinking (yes/no) was considered as one of the components of the selected indicator of following alcohol-related recommendations. In the high-income countries, it is common that poorer people are more likely to be abstainers than richer people and that those less affluent, on average, drink less frequently than the more affluent individuals [3,11,26,27,28]. However, HED is more common in poorer societies [3,11,27].

One traditional differentiation in alcohol consumption, also observed in our study, has been pointed out with regard to sex. Men generally drink considerably more alcohol than women, in terms of frequency and the volume of drinking [29]. Within a given society, sex differentiation is often greater among poorer people than among the richer ones [3]. Burden of alcohol-attributable diseases also varies by sex (with men at a higher risk than women) [3]. This can be explained by different drinking patterns (as described above) and by the fact that men are usually representing other risky behaviours (tobacco smoking, unhealthy diet, and obesity) more frequently than women [3,30,31,32,33].

We found higher odds of not following recommendations related to alcohol consumption for the subjects declaring having a permanent or a temporary job compared to those unemployed ones. In general, the study population is a socially disadvantaged population. However, within this group, some part of the participants was employed (with a salary not exceeding minimum income stated by The Social Welfare Act to be able to receive social aid). It needs to be pointed out that the educational level of the study participants was much lower than that of the general population in Poland (as an example, higher education was indicated by 5.4% of the study participants, whereas, based on a Demographic Yearbook of Poland, 2016, 24.3% Poles represented a higher educational level) [34]. Moreover, when considering characteristics of the district (more than 90% of its residents representing rural areas), the majority of people declaring having a permanent or temporary job were working in agriculture or related professions and were mostly blue-collar workers [15,16,17]. A study that examined the relationship between country-level characteristics and individual socio-economic status (SES), and individual alcohol consumption in 33 countries, has shown that, for both sexes and all countries, higher individual SES was positively associated with their drinking status [10]. In many societies, access to alcoholic beverages is greater for those representing a more affluent status or among employed individuals [3,35,36]. Higher percentages of risky drinking observed among the people with a permanent or a temporary job in our study as compared to the unemployed ones can be interpreted by the amount of money that the participants needed to have to be able to spend it on alcohol. The existing studies point out that risky drinking is often more prevalent among blue-collar workers [3]. Moreover, alcohol consumption can be influenced by job/workplace characteristics, including employee dissatisfaction, workplace control, and workplace culture [5,37,38,39,40,41,42,43]. However, this was not evaluated in the current study. It can be a subject of more in-depth analyses in the future.

Our analyses indicate that the individuals who declared unhealthy lifestyle-related behaviours at the same time did not comply with recommendations on alcohol consumption. Other studies have also found similar associations, suggesting the potential for clustering behaviours acting synergistically toward individual health [1,44,45]. As an example, in the study by Sandoval et al., high health conscious individuals (representing those with a high health consciousness index based on dietary habits, physical activity, and smoking status) including both men and women were less likely to consume alcohol regularly and engage in HED [44].

Individuals with low SES experience disproportionately greater alcohol-attributable health consequences than individuals with high SES [1]. Differential alcohol use patterns, vulnerability, presence of other lifestyle-related factors, and access to healthcare or interventional facilities among socio-economic groups can explain socio-economic inequalities in alcohol-related harm [1,9,10,12,46,47,48].

Alcohol consumption as one of the main factors for death, disease, and disability has substantial financial burden on a society, with economic costs ranging from 1.3% to 3.3% of the gross domestic product in the middle-income and high-income countries [49]. Thus, effective interventions tailored to the specific needs represented by a society are crucial for changing the existing prevalence and patterns of alcohol use. The existing studies point out that initiatives addressing neighborhood planning, zoning, and licensing are among the most effective approaches toward reducing socio-economic inequalities in alcohol consumption [5]. Minimum unit pricing has been indicated as such a strategy, which should mostly affect heavy drinkers [50,51]. Additionally, screening and brief intervention have been shown to be an effective approach to identify and reduce risky alcohol use [52,53]. Interventions should not only focus on alcohol consumption but also on other lifestyle-related factors. They should eliminate smoking and promote a healthy diet, recreational physical activity, or recommended BMI. Given that individuals with low SES are less likely to utilize primary care services, equal access to screening and intervention facilities would be a prerequisite for a strategy to reduce socio-economic inequalities [1].

The current analyses were conducted among socially-disadvantaged adults, which constitutes the major strength of the study. To the best of our knowledge, such comprehensive analyses have been performed for the first time among such vulnerable populations in Poland. Other advantages of this study are related to a medium-to-large sample size, the substantial participation rate, and face-to-face interviews selected to obtain relevant data. As pointed out above, another strength of this study is related to the fact that we have assessed correlates of not following alcohol-related recommendations (in one indicator we considered the level and pattern of alcohol consumption), whereas other studies rely mostly on a single indicator (volume of alcohol or frequency of drinking or HED).

The limited generalizability of the findings needs to mentioned (as the study was conducted among social assistance beneficiaries from a socio-economically disadvantaged rural area in Poland). Additionally, a complex indicator of alcohol consumption may limit direct comparisons with other studies in this field. Moreover, causality cannot be inferred due to the cross-sectional nature of the current study. Finally, measurement of alcohol use is also a potential limitation of the presented analyses since some underreporting could occur.

## 5. Conclusions

Our study indicates that being men, having a permanent or a temporary job, and coexistence of other unfavorable lifestyle-related factors are important correlates of not following recommendations for alcohol consumption among beneficiaries of government welfare assistance. There is still a need for in-depth studies on the factors influencing decisions related to alcohol consumption among socially-disadvantaged individuals. Considering not fully conclusive results, more research should focus on the employment status as a correlate of unhealthy alcohol use as well as going beyond available financial resources to buy such products. With this regard, workplace characteristics and social functioning should be an urgent interest.

Taking into account that high proportion of this vulnerable population does not follow existing recommendations, development and implementation of effective interventions aimed at reduction of alcohol consumption is still a necessity. Nevertheless, because of a complex and diverse nature of social determinants that have an influence on different populations, it is impossible to point to one strategy that could result in a reduction of alcohol consumption and relate to it adverse effects on a community-wide basis. Therefore, it is vital to use the most relevant and reliable information to elaborate a set of measures that would be best for individual groups and specific settings. To do that, we have to understand the ways in which alcohol negatively influences various groups. Additionally, since unfavourable lifestyle factors coexist and affect alcohol consumption, interventions to address them are necessary.

## Figures and Tables

**Table 1:** Characteristics of the study population.

Variables	Total * n = 1644 100%	Men *n* = 545 (33.2%)		Women *n* = 1099 (66.8%)		p **
		Total *n* (%)	Not Following Alcohol-Related Recommendations *n* = 364 (66.8%)	Total *n* (%)	Not Following Alcohol-Related Recommendations *n* = 330 (30.0%)	
**Age (years)**						
<30	181 (11.0%)	42 (23.2%)	28 (66.7%)	139 (76.8%)	46 (33.1%)	*p* < 0.001
30–39	700 (42.6%)	195 (27.9%)	134 (68.7%)	505 (72.1%)	148 (29.3%)	*p* < 0.001
40–49	557 (33.9%)	203 (36.4%)	133 (65.5%)	354 (63.6%)	102 (28.8%)	*p* < 0.001
50–59	206 (12.5%)	105 (51.0%)	69 (65.7%)	101 (49.0%)	34 (33.7%)	*p* < 0.001
**Education**						
Primary	443 (27.0%)	191 (43.1%)	132 (69.1%)	252 (56.9%)	77 (30.6%)	*p* < 0.001
Vocational	549 (33.4%)	221 (40.3%)	139 (62.9%)	328 (59.7%)	104 (31.7%)	*p* < 0.001
Secondary	562(34.2%)	125 (22.2%)	87 (69.6%)	437 (77.8%)	115 (26.3%)	*p* < 0.001
High	90 (5.4%)	8 (8.9%)	6 (75.0%)	82 (91.1%)	34 (41.5%)	*p* > 0.05
**Employment status**						
Permanent job	492 (29.9%)	210 (42.7%)	142 (67.6%)	282 (57.3%)	83 (29.4%)	*p* < 0.001
Temporary job	140 (8.5%)	64 (45.7%)	51 (79.7%)	76 (54.3%)	29 (38.2%)	*p* < 0.001
Disabled or retired	53 (3.2%)	27 (50.9%)	14 (51.9%)	26 (49.1%)	9 (34.6%)	*p* > 0.05
Unemployed	959 (58.3%)	244 (25.4%)	157 (64.3%)	715 (74.6%)	209 (29.2%)	*p* < 0.001
**Subjective assessment of monthly income**						
Sufficient to cover all living needs plus may save a certain amount	19 (1.2%)	4 (21.1 %)	4 (100.0%)	15 (78.9%)	6 (40.0%)	*p* = 0.05
Sufficient to cover all living needs	182 (11.1%)	52 (28.6%)	35 (67.3%)	130 (71.4%)	39 (30.0%)	*p* < 0.001
Sufficient to cover basic needs only	867 (52.7%)	267 (30.8%)	175 (65.5%)	600 (69.2%)	172 (28.7%)	*p* < 0.001
Not sufficient to cover even basic needs	411 (25.0%)	173 (42.1%)	121 (69.9%)	238 (57.9%)	84 (35.3%)	*p* < 0.001
Difficult to say	165 (10.0%)	49 (29.7%)	29 (59.2%)	116 (70.3%)	29 (25.0%)	*p* < 0.001
**Subjective assessment of living conditions**						
Fair or rather fair	763 (46.4%)	223 (29.2%)	138 (61.9%)	540 (70.8%)	157 (29.1%)	*p* < 0.001
Neither fair nor poor	744 (45.3%)	272 (36.6%)	190 (69.9%)	472 (63.4%)	147 (31.1%)	*p* < 0.001
Rather poor	79 (4.8%)	27 (34.2%)	22 (81.5%)	52 (65.8%)	16 (30.8%)	*p* < 0.001
Very poor	26 (1.6%)	13 (50.0%)	8 (61.5%)	13 (50.0%)	6 (46.2%)	*p* > 0.05
Difficult to say	32 (2.0%)	10 (31.3%)	6 (60.0%)	22 (68.8%)	4 (18.2%)	*p* < 0.03
**Cohabitation with partner and/or family**						
Yes	1389 (84.5%)	462 (33.3%)	303 (65.6%)	927 (66.7%)	270 (29.1%)	*p* < 0.001
No	255 (15.5%)	83 (32.6%)	61 (73.5%)	172 (67.4%)	60 (34.9%)	*p* < 0.001
**Children <15 years**						
Yes	1112 (67.6%)	366 (32.9%)	234 (63.9%)	746 (67.1%)	216 (28.9%)	*p* < 0.001
No	532 (32.4%)	179 (33.6%)	130 (72.6%)	353 (66.4%)	114 (32.3%)	*p* < 0.001
**Subjective assessment of life satisfaction**						
Extremely satisfied or satisfied	678 (41.2%)	207 (30.5%)	131 (63.3%)	471 (69.5%)	133 (28.2%)	*p* < 0.001
Neutral	819 (49.8%)	276 (33.7%)	193 (69.9%)	543 (66.3%)	166 (30.6%)	*p* < 0.001
Slightly dissatisfied	101 (6.1%)	38 (37.6%)	25 (65.8%)	63 (62.4%)	22 (34.9%)	*p* < 0.003
Dissatisfied or extremely dissatisfied	46 (2.8%)	24 (52.2%)	15 (62.5%)	22 (47.8%)	9 (40.9%)	*p* > 0.05
**Subjective health state**						
Fair/rather fair	1075 (65.4%)	323 (30.0%)	224 (69.4%)	752 (70.0%)	228 (30.3%)	*p* < 0.001
Neither fair nor poor	393 (23.9%)	141 (35.9%)	88 (62.4%)	252 (64.1%)	71 (28.2%)	*p* < 0.001
Rather poor/poor	176 (10.7%)	81 (46.0%)	52 (64.2%)	95 (54.0%)	31 (32.6%)	*p* < 0.001
**Number of health problems**						
0	221 (13.7%)	99 (44.8%)	62 (62.6%)	122 (55.2%)	33 (27.0%)	*p* < 0.001
1–3	863 (53.6%)	297 (34.4%)	204 (68.7%)	566 (65.6%)	169 (29.9%)	*p* < 0.001
4–6	432 (26.8%)	115 (26.6%)	76 (66.1%)	317 (73.4%)	93 (29.3%)	*p* < 0.001
≥ 7	95 (5.9%)	27 (28.4%)	17 (63.0%)	68 (71.6%)	23 (33.8%)	*p* < 0.01
**Following smoking-related recommendations**						
Yes	1039 (63.3%)	259 (24.9%)	165 (63.7%)	780 (75.1%)	214 (27.4%)	*p* < 0.001
No	603 (36.7%)	285 (47.3%)	198 (69.5%)	318 (52.7%)	115 (36.2%)	*p* < 0.001
**Following diet-related recommendations**						
Yes	157 (9.6%)	43 (27.4%)	30 (69.8%)	114 (72.6%)	30 (26.3%)	*p* < 0.001
No	1487 (90.4%)	502 (33.8%)	334 (66.5%)	985 (66.2%)	300 (30.5%)	*p* < 0.001
**Following recommendations related to recreational physical activity**						
Yes	424 (26.2%)	135 (31.8%)	90 (66.7%)	289 (68.2%)	84 (29.1%)	*p* < 0.001
No	1194 (73.8%)	402 (33.7%)	268 (66.7%)	792 (66.3%)	238 (30.1%)	*p* < 0.001
**Following BMI related recommendations**						
Yes	697 (42.4%)	196 (28.1%)	138 (70.4%)	501 (71.9%)	148 (29.5%)	*p* < 0.001
No	947 (57.6%)	349 (36.8%)	226 (64.8%)	598 (63.2%)	182 (30.4%)	*p* < 0.001
**Combined HLI**						
0	291 (18.0%)	144 (49.5%)	102 (70.8%)	147 (50.5%)	52 (35.4%)	*p* < 0.001
1	646 (40.0%)	210 (32.5%)	131 (62.4%)	436 (67.5%)	133 (30.5%)	*p* < 0.001
2	406 (25.1%)	128 (31.5%)	83 (64.8%)	278 (68.5%)	81 (29.1%)	*p* < 0.001
3	255 (15.8%)	51 (20.0%)	39 (76.5%)	204 (80.0%)	50 (24.5%)	*p* < 0.001
4	18 (1.1%)	3 (16.7%)	2 (66.7%)	15 (83.3%)	5 (33.3%)	*p* > 0.05

* The numbers might not sum up to the total sample as some missing data could occur. ** Men vs. women not following alcohol-related recommendations. *p*-values was calculated using the test for equality of two fractions. HLI—Healthy Lifestyle Index (as the sum of points given for each of the four lifestyle-related factors (smoking, BMI, diet, and recreational physical activity). Participants received 1 if they followed the relevant recommendations and 0 if the recommendations were not followed). BMI—Body Mass Index.

**Table 2:** Odds ratio (OR) and 95% confidence interval (CI) for not following recommendations for alcohol consumption by socio-demographic and lifestyle-related characteristics of the population.

Variables	Unadjusted Model		Adjusted Model	
	OR (95% CI)	*p*	OR (95% CI)	*p*
Sex				
Man	4.69 (3.76–5.84)	<0.001	4.49 (3.52–5.68)	< 0.001
Women	1		1	
**Age (years)**				
< 30	1		1	
30–39	0.98 (0.70–1.36)	0.88	0.88 (0.61–1.27)	0.50
40–49	1.03 (0.75–1.48)	0.76	0.78 (0.53–1.14)	0.20
50–59	1.45 (0.97–2.16)	0.07	0.84 (0.53–1.14)	0.45
**Education**				
Primary	1.51 (1.18–1.93)	<0.001	1.03 (0.77–1.37)	0.85
Vocational	1.35 (1.07–1.70)	0.01	0.99 (0.76–1.29)	0.93
Secondary or higher	1		1	
**Employment status**				
Permanent or temporary job	1.51 (1.23–1.85)	<0.001	1.23 (1.01–1.63)	0.04
Disabled or retired	1.24 (0.71–2.17)	0.45	0.88 (0.48–1.63)	0.69
Unemployed	1		1	
**Subjective assessment of monthly income**				
Sufficient to cover all living needs	1		1	
Sufficient to cover basic needs only	0.93 (0.68–1.27)	0.65	0.98 (0.73–1.33)	0.68
Not sufficient to cover even the basic needs	1.39 (0.99–1.95)	0.06	1.05 (0.78–1.65)	0.30
Difficult to say	0.76 (0.49–1.15)	0.20	0.69 (0.43–1.11)	0.13
**Subjective assessment of living conditions**				
Fair or rather fair	1		1	
Neither fair nor poor or difficult to say	1.28 (1.05–1.57)	0.02	1.13 (0.89–1.45)	0.32
Rather poor or very poor	1.56 (1.03–2.34)	0.03	1.35 (0.82–2.21)	0.23
**Cohabitation with partner and/or family**	1		1	
Yes	1		1	
No	1.29 (0.98–1.68)	0.07	1.22 (0.84–1.77)	0.31
**Children <15 years**				
Yes	1		1	
No	1.25 (1.02–1.54)	0.04	1.13 (0.85–1.52)	0.40
**Subjective assessment of life satisfaction**				
Extremely satisfied/satisfied	1		1	
Neutral	1.22 (0.99–1.51)	0.06	1.10 (0.86–1.42)	0.45
Slightly dissatisfied	1.36 (0.90–2.08)	0.15	1.07 (0.65–1.77)	0.80
Dissatisfied/extremely dissatisfied	1.72 (0.94–3.11)	0.08	1.06 (0.53–2.13)	0.87
**Subjective health state**				
Fair/rather fair	1			
Neither fair nor poor	0.94 (0.74–1.18)	0.58		
Rather poor/poor	1.23 (0.89–1.69)	0.20		
**Number of health problems**				
0	1.04 (0.64–1.69)	0.52		
1–3	1.05 (0.68–1.61)	0.47		
4–6	0.88 (0.56–1.39)	0.33		
≥ 7	1			
**Combined HLI**				
0–2	1.41 (1.08–1.85)	0.01	1.11 (1.01–1.49)	0.04
3–4	1		1	

HLI—Healthy Lifestyle Index (as the sum of points given for each of the four lifestyle-related factors (smoking, BMI, diet, and recreational physical activity). Participants received 1 if they followed the relevant recommendations and 0 if the recommendations were not followed).

## Supplementary Materials

The following are available online at https://www.mdpi.com/1660-4601/17/23/9074/s1, Table S1. Odds ratio (OR) and 95% confidence interval (CI) for not following recommendations for alcohol consumption by socio-demographic and lifestyle-related characteristics of the population—analysis stratify by gender.
