# Supplementary material for: Correlates of Alcohol Consumption Among a Socially-Disadvantaged Population in Poland
Source: Int J Environ Res Public Health. 2020 Dec 4;17(23):9074. doi: 10.3390/ijerph17239074 (PMC7730564; doi:10.3390/ijerph17239074)
Supplement: Supplementary file 1 [file ijerph-17-09074-s001.pdf]

Correlates of alcohol consumption among a socially-disadvantaged population in Poland

Kinga Polanska* and Dorota Kaleta

Supplementary Materials

Table S1. Odds ratio (OR) and 95% confidence interval (CI) for not following recommendations for alcohol consumption by socio-demographic and lifestyle-related characteristics of the population – analysis stratify by gender.

Variables	Men				Women			
	Unadjusted Model		Adjusted Model		Unadjusted Model		Adjusted Model*	
	OR (95% CI)	*p*	OR (95% CI)	*p*	OR (95% CI)	*p*	OR (95% CI)	*p*
**Age (years)**								
< 30	1				1			
30–39	1.10 (0.54–2.24)	0.80			0.84 (0.56–1.25)	0.39		
40–49	0.95 (0.47–1.92)	0.89			0.82 (0.54–1.25)	0.35		
50–59	0.96 (0.45–2.05)	0.91			1.03 (0.60–1.77)	0.93		
**Education**								
Primary	0.96 (0.59–1.56)	0.88			1.09 (0.79–1.52)	0.60		
Vocational	0.73 (0.46–1.16)	0.18			1.15 (0.85–1.56)	0.35		
Secondary or higher	1				1			
**Employment status**								
Permanent or temporary job	1.32 (0.91–1.91)	0.14			1.10 (0.84–1.45)	0.49		
Disabled or retired	0.60 (0.27–1.33)	0.21			1.28 (0.56–2.92)	0.55		
Unemployed	1				1			
**Subjective assessment of monthly income**								
Sufficient to cover all living needs	1				1			
Sufficient to cover basic needs only	0.83 (0.44–1.55)	0.57			0.89 (0.60–1.32)	0.57		
Not sufficient to cover even the basic needs	1.01 (0.53–1.95)	0.96			1.21 (0.78–1.88)	0.40		
Difficult to say	0.63 (0.28–1.41)	0.26			0.74 (0.42–1.28)	0.28		
**Subjective assessment of living conditions**								
Fair or rather fair	1		1		1			
Neither fair nor poor or difficult to say	1.40 (0.97–2.04)	0.07	1.39 (0.96–2.01)	0.08	1.07 (0.82–1.40)	0.60		
Rather poor or very poor	1.85 (0.86–3.98)	0.12	1.77 (0.82–3.83)	0.15	1.25 (0.72–2.16)	0.43		
**Cohabitation with partner and/or family**								
Yes	1				1			

No	1.45 (0.86–2.46)	0.16			1.30 (0.92–1.84)	0.13
**Children < 15 years**						
Yes	1		1		1	
No	1.50 (1.01–2.22)	0.04	1.46 (0.99–2.17)	0.06	0.17 (0.89–1.54)	0.26
**Subjective assessment of life satisfaction**						
Extremely satisfied/Satisfied	1				1	
Neutral	1.35 (0.92–1.98)	0.13			1.12 (0.85–1.47)	0.42
Slightly dissatisfied	1.12 (0.54–2.31)	0.77			1.36 (0.78–2.38)	0.27
Dissatisfied/Extremely dissatisfied	0.97 (0.40–2.32)	0.94			1.76 (0.73–4.22)	0.21
**Subjective health state**						
Fair/rather fair	1				1	
Neither fair nor poor	0.73 (0.48–1.11)	0.14			0.90 (0.66–1.24)	0.52
Rather poor/poor	0.79 (0.47–1.32)	0.37			1.11 (0.71–1.76)	0.65
**Number of health problems**						
0	0.99 (0.41–2.38)	0.97			0.73 (0.38–1.38)	0.33
1–3	1.29 (0.57–2.93)	0.54			0.83 (0.49–1.42)	0.50
4–6	1.15 (0.48–2.75)	0.76			0.81 (0.46–1.42)	0.47
>7	1				1	
**Combined HLI**						
0–2	0.60 (0.31–1.16)	0.13			1.33 (0.95–1.87)	0.09
3–4	1				1	

HLI—Healthy Lifestyle Index (as the sum of points given for each of the four lifestyle-related factors (smoking, BMI, diet, recreational physical activity); participants received 1 if they followed the relevant recommendations and 0 if the recommendations were not followed). * Multivariable model was not performed as only for combined HLI the requirements for inclusion into multivariable analysis ($p < 0.1$) was achieved.
